# The Mallet

**Published:** 1873-06

**Authors:** S. E. Goe


### THE MALLET.

BY S. E. GOE.

What has become of the mallet ? Our dental journals are certainly very reticent on the subject. Dental associations no longer put it into their lists of subjects for discussion, obscure country practitioners who stay at home might easily suppose the instrument had been dropped and was no longer in use. After its introduction as a means of applying force for condensing gold into cavities of decay, it was much talked about by leading members of the profession every where, it was a constant topic of discussion in dental societies, and headed articles in nearly every issue of the dental journals. Has the subject been exhausted, or has the mallet yielded up all its secrets to professional investigation ?

I think not, and would like to see the subject opened once more to free discussion. It is highly probable that the period of silence on this topic through which we are passing, may have witnessed the development of new methods of applying this instrument, the discovery of new material for its construction, and an accumulation of valuable suggestions as to its use. If these could be opened up to the profession it is not unlikely that our later experience would show that progress has been made ; and it might bring forth new and entirely unexpected results.

Since it is only by thorough investigation and the exchange of views that the profession is elevated, it is or should be the duty of every member in it to do what he can in that direction. In pursuance of the above thought I propose giving in as few words as possible, what seems to me to be the best shape, size, and materials for constructing the mallet and the best methods of using it.

The shape of the mallet should be cylindrical. Its length being greater than its diameter. There is an object in giving the instrument this shape, as there is greater uniformity of force given to the blow in consequence of its striking the plugging instrument at a point nearer the center of the weight. There is a nice point to be observed, too, in shaping the handle of the mallet. This should be flattened, its flatence surfaces being parallel with the longitudinal axis of mallet. It can there be held firmly in the hand without turning. This is a point worth observing especially if the operator is using the mallet himself. He is enabled to determine whether or not the instrument is in the proper position without taking the eyes from the point of the instrument in the cavity.

The size of the mallet should be determined by the weight desired. There is diversity of opinion in regard to what the weight should be. Some practitioners advise the use of a mallet weighing sixteen ounces, whilst others regard a mallet weighing four ounces as sufficiently heavy. So far as my experience teaches me, a mallet weighing seven or eight ounces will give the best results ; yet there may be cases where a lighter mallet may be used with good effect; one and three-eighths inches in length by one and one-eighth in diameter would be the dimensions of a mallet weighing eight ounces, if made of lead.

As to the material for constructing mallets, lead undoubtedly possesses more advantages than any other. Almost every available substance has been tested, but nothing has superceded lead. The method of constructing a lead mallet is quite simple. We have only to make a band of German silver (or any substance that will admit of being worked in that manner), pour melted lead into it, drill a hole with an ordinary beveled point drill for the reception of the handle, and the mallet is made. The handle may be shaped and fastened in without trouble.

A favorite method of using the mallet with many, is to take it in their own hand,—in all cases that will admit of it. It requires but little practice to learn to handle both the mallet and the instrument, and it is certainly a very satisfactory way of using it. The operator is enabled to use it with greater accuracy than it is possible for any assistant to acquire. But it is sometimes necessary to have an assistant to use the mallet. Then we come to another important consideration. What are the requirements of a malleter? Too much care and judgment cannot be exercised in selecting a malleter. The assistant while malleting should not be looking out of the window or at objects about the room, and paying little attention to the part of the work he is to perform, as it is the case with many.

The maleter must be a person of quick perception, one who can interpret signs made by the operator instinctively. A sluggish, awkward person should never stand beside the patient, oftentimes rapidity of action as well as delicacy of movement is required of the assistant.

The assistant should be neat and tidy about his person and of good manners. It is very annoying to most patients to have the malleter looking down their throats whilst they are malleting, this they should be made to understand. Some patients object to having a third person about at all, whilst they are in the chair, therefore it is important that the malleter know how to avoid offending sensitive persons.

It is essential to etiquette in the assistant not to sneeze or cough while beside the patient. If this is necessary they should retire for the time being!

These last few hints may not bear directly upon the use of the mallet, yet they are so closely allied to it that they are worthy of mention in this connection.

I have not thought of treating the subject of using the mallet exhaustively, or giving all directions to the assistant. Much more might be said. I would be glad to have some one come out with a series of articles on the subject. They would not only be instructive but might start others to thinking over the matter, and a great deal might be done towards perfecting the mallet and the methods of its application.

The general behavior of the assistant, making unpleasant remarks, looking into the mouth of the patient and speaking at the same time, shocking the olfactories of the patient with a bad breath are subjects that might be treated more fully than has been done in this paper.

---

A writer has said that the summit of human happiness was the possession of a good digestion. Trollope says that the possession of good digestion is to be in Paradise. That being the case, a person with offensive teeth is beyond the pale of Paradise, and the growth of good dentistry heralds the millenial day. F.

